# Fatal encephalitis caused by Shamonda virus Europe 1 (SHAV-EU1) in a patient with a severely weakened immune system, France, 2026

**DOI:** 10.2807/1560-7917.ES.2026.31.42.2600883

**Published:** 2026-10-22

**Authors:** Margot Bonnet, Hélène Labussière-Wallet, Pauline Dumez, Lucie Jauffret, Lyderic Aubert, Aurélie Avondo-Ray, Carole Seurret, Perrine Devic, Sabrine Souci, Syria Laperche, Léa Saint Martin, Emilie Frobert, Alexandre Gaymard, Jennifer Giorgi, Quentin Semanas, Richard Chalvignac, Antoine Oblette, Audrey Lapendry, Bruno Pozzetto, Thomas Perpoint, Mikail Kelleci, Aurélie Sécula, Charlotte Foret-Lucas, Clement Javaux, Florence Morfin, Antonin Bal, Elise Brottet, Catarina Krug, Frederic Jourdain, David Meyronet, Florence Ader, Jean-Luc Guérin, Laurence Josset, Grégory Destras

**Affiliations:** 1 Anesthésie–Réanimation–Médecine Intensive, Hospices Civils de Lyon, Hôpital Lyon Sud, Pierre Benite, Lyon, France; 2 Service d'Hématologie clinique, Hospices Civils de Lyon, Hôpital Lyon Sud, Pierre Benite, Lyon, France; 3 Service de Neurologie, Hospices Civils de Lyon, Hôpital Lyon Sud, Pierre Benite, Lyon, France; 4 Department of Medical Oncology, Hôpitaux Nord Ouest, Villefranche, France; 5 Direction Générale de la Santé, Paris, France; 6 Établissement Français du Sang, La Plaine Saint Denis, France; 7 Service de Virologie, Plateforme de séquençage Genepii, Centre National de Référence des Virus Respiratoires, Hospices Civils de Lyon, Groupement Hospitalier Nord, Croix-Rousse, Lyon, France; 8 CIRI, Centre International de Recherche en Infectiologie, Team CARVI, Univ Lyon, Inserm, U1111, Université Claude Bernard Lyon 1, CNRS, UMR5308, ENS de Lyon, France; 9 Centre International de Recherche en Infectiologie (CIRI), Team GIMAP, Inserm U1111, CNRS UMR5308, ENS de Lyon, Université Claude Bernard - Lyon 1, Université Jean Monnet de Saint-Etienne, Saint-Etienne, France, & Haut Conseil de la Santé Publique, Paris, France; 10 Department of Infectious Diseases and Tropical Medicine, Hôpital de la Croix-Rousse, Hospices Civils de Lyon, Lyon, France; 11 Université Toulouse, ENVT, INRAE, IHAP, Toulouse, France; 12 Santé publique France, Lyon/Saint Maurice, France; 13 Department of Pathology and Neuropathology, Groupe Hospitalier Est, Hospices Civils de Lyon, 59 Boulevard Pinel, 69500 Bron, France; Cancer Research Centre of Lyon (CRCL) INSERM 1052, CNRS 5286, 28 Rue Laennec, 69008 Lyon, France; Faculty of Medicine of Lyon, Université Claude Bernard Lyon 1, Lyon, France; 14 Centre International de Recherche en Infectiologie (CIRI), Inserm 1111, Université Claude Bernard Lyon 1, CNRS, UMR5308, École Normale Supérieure de Lyon, Univ, Lyon F-69007, France

**Keywords:** Orthobunyavirus, Shamonda virus, encephalitis, metatranscriptomics

## Abstract

New Shamonda virus clades (SHAV-EU1 and SHAV-EU2) emerged in Europe in summer 2026, primarily affecting cattle. We report the first human infection with SHAV-EU1: a French patient with a severely weakened immune system developed fatal encephalitis in September 2026, 1 month after their allogeneic stem cell transplantation. Metatranscriptomics detected SHAV-EU1 in cerebrospinal fluid, confirmed by RT-PCR. Biopsies from central nervous system showed high viral loads. Investigations revealed SHAV-EU1 in serum, respiratory and stool samples at least for 2 months before encephalitis onset.

Shamonda virus (SHAV) is an enveloped, negative-sense, single-stranded RNA virus with a tri-segmented genome, a member of the Simbu serogroup within the *Orthobunyavirus* genus [1]. This virus, presently considered by the International Committee on Taxonomy of Viruses (https://ictv.global/) to be a variant of the *Orthobunyavirus schmallenbergense *species, was detected in Europe for the first time in July 2026 in cattle. Two variants have recently emerged through reassortment, involving ancestral Shamonda strains: SHAV-EU1 [2,3], detected in France, southern Germany, Switzerland, Austria, Italy, Spain and Portugal; and SHAV-EU2 [4], detected in north-western Germany, the Netherlands and north-western France [5]. Transmission is thought to occur mainly through *Culicoides* biting midges [6], with no evidence of direct animal-to-animal transmission, except for transplacental transmission. Herein we report initial investigations into the first documented case of human infection caused by SHAV.

## Clinical presentation

On 23 September 2026 (day (D)0), a patient in their sixties developed acute cortical blindness and confusion, with normal brain magnetic resonance imaging (MRI) and cerebrospinal fluid (CSF) examination. This patient had a history of T-cell prolymphocytic leukaemia diagnosed in November 2025. They underwent first-line treatment with alemtuzumab (Campath) from December 2025 to February 2026, followed by an allogeneic haematopoietic stem cell transplantation (HSCT) on D−28 from a human leukocyte antigen (HLA) matched related donor. The conditioning regimen consisted of thiotepa, fludarabine and busulfan and the graft vs host disease (GVHD) prophylaxis consisted of post-transplant cyclophosphamide and cyclosporine. The patient lived in a rural area in the French Rhône-Alpes region. They had been hospitalised in Lyon continuously since 18 August 2026, corresponding to D−36 (Figure 1).

**Figure 1:**
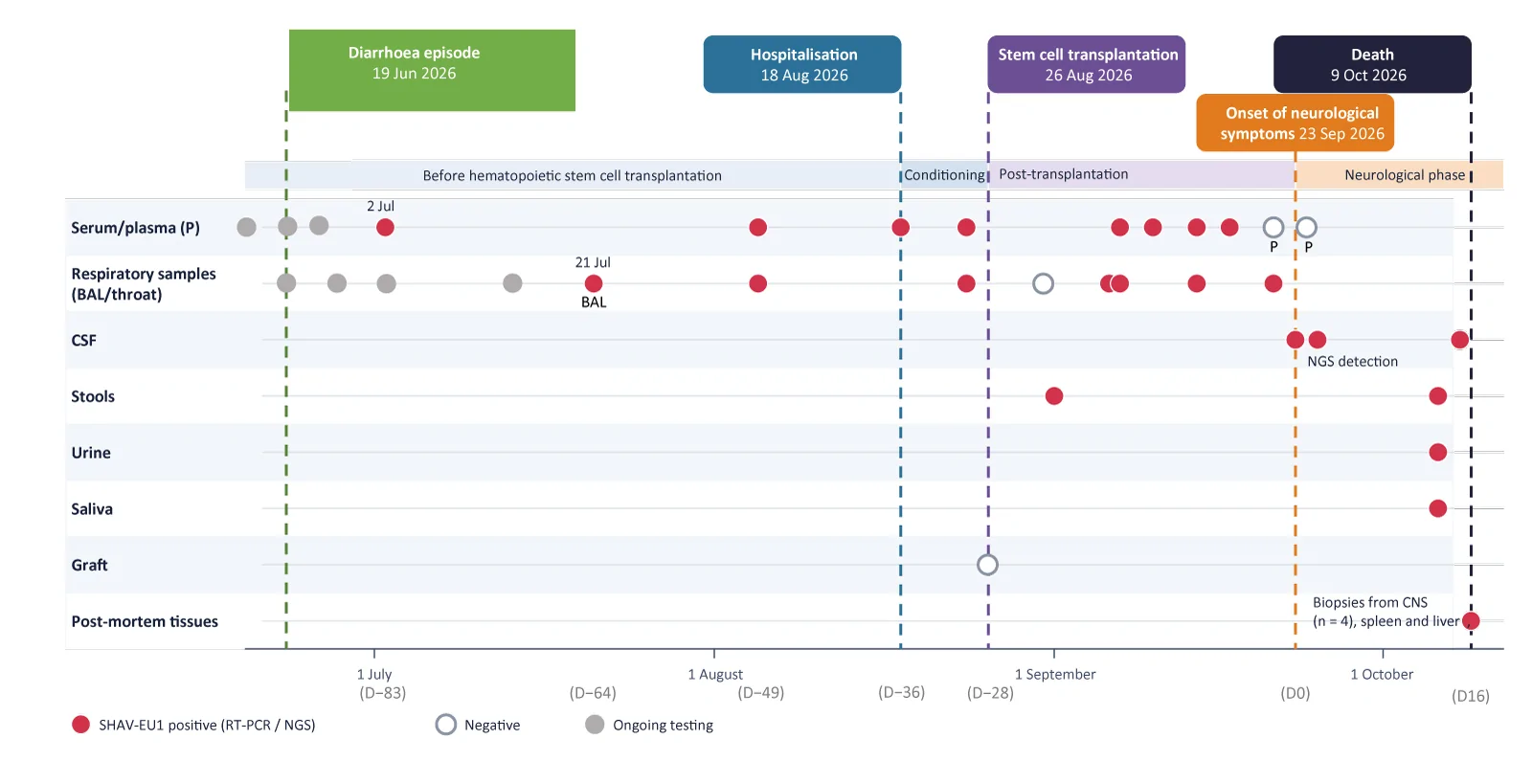
Timeline of detection of Shamonda virus Europe 1 in samples from a patient, France, 2026

BAL: bronchoalveolar lavage; CNS: central nervous system; Cq: quantification cycle; D: day; D0: onset of the neurological symptoms; NGS: next-generation sequencing; SHAV-EU1: Shamonda virus Europe 1.

Each phase of the patient’s clinical history is depicted, with dots representing all patient samples retrospectively tested for SHAV-EU1 by RT-PCR at the hospitals. All Cq values are presented in the Table.

Fever appeared on D1, followed by meningism, pyramidal signs, seizures and coma requiring intubation and mechanical ventilation. Circulating CD19 + and CD20 + B-cells were undetectable, with hypogammaglobulinaemia. Repeat MRI on 30 September showed extensive bilateral grey matter abnormalities. No metabolic, nor haemodynamic cause was identified. The patient showed no clinical improvement despite empirical intravenous cefotaxime 200 mg/kg/day, amoxicillin 200 mg/kg/day and aciclovir 15 mg/kg every 8 h and high-dose corticosteroids. Given the persistent comatose status, the absence of brainstem reflexes and the severity of the MRI lesions, life-sustaining treatment was withdrawn following in-depth consultations with the family and within the care team. The patient passed away on 9 October 2026, 16 days after the onset of the neurological symptoms. Detailed clinical, neuroimaging and neuropathological findings will be reported separately.

## Microbiological testing

Two lumbar punctures were performed on D0 and D2. Analysis of CSF showed no clear inflammatory profile, with leukocyte counts of 4 and 6 cells/mm^3^ (norm: <  2 cells/mm^3^), respectively, and a predominance of neutrophils. Despite extensive investigations, no aetiological diagnosis was initially established through routine diagnostic testing (Table).

**Table:** Virological testing of samples from a patient for Shamonda virus infection, France, 2026

Day (according to neurological symptoms onset)	Sample type	Date	Sample ID	Cq SHAV-EU1	Cq ADIALYO SHLV
D − 83	Serum	2 Jul	SHAV-LYON16	33.43	29.67
D − 64	BAL	21 Jul	SHAV-LYON23	37.03	35.24
D − 49	Serum	5 Aug	SHAV-LYON17	37.38	33
	Nose/throat respiratory sample	5 Aug	SHAV-LYON24	ND	34.77
D − 36	Serum	18 Aug	SHAV-LYON18	ND	33.57
D − 30	Serum	24 Aug	SHAV-LYON19	36.06	32.97
	Throat respiratory sample	24 Aug	SHAV-LYON25	36.32	34.39
D − 28	Transplant (stem cells)	26 Aug	SHAV-LYON08	ND	ND
D − 23	Throat respiratory sample	31 Aug	SHAV-LYON26	36.46	ND
D − 21	Stool 2	2 Sep	SHAV-LYON30	34.94	30.8
D − 17	Serum	7 Sep	SHAV-LYON20	26.69	23.25
	Oral respiratory sample	6 Sep	SHAV-LYON27	34.43	31.27
D − 16	Throat respiratory sample	7 Sep	SHAV-LYON28	32.5	30.13
D − 13	Serum	10 Sep	SHAV-LYON21	28.08	25.95
D − 9	Serum	14 Sep	SHAV-LYON22	31.26	28.74
	Throat respiratory sample	14 Sep	SHAV-LYON29	37.54	32.27
D − 6	Plasma	17 Sep	SHAV-LYON03	ND	34.06
D − 2	Oral respiratory sample	21 Sep	SHAV-LYON31	34.70	33.44
D0	CSF 1	23 Sep	SHAV-LYON01	37.39	33.06
D1	Plasma	24 Sep	SHAV-LYON04	34.65	ND
D2	CSF 2	25 Sep	Detection by metatranscriptomics		
D7	Plasma	30 Sep	SHAV-LYON05	ND	ND
D14	Stool 1	6 Oct	SHAV-LYON02	29.03	29.33
	Saliva	6 Oct	SHAV-LYON06	25.24	24.18
	Urine	6 Oct	SHAV-LYON07	28.89	ND
D15	CSF 3	7 Oct	SHAV-LYON15	31.58	29.03
D17	Cerebellum biopsy	9 Oct	SHAV-LYON09	17.14	13.91
	Frontal biopsy of brain	9 Oct	SHAV-LYON10	17.57	13.85
	Occipital biopsy of brain	9 Oct	SHAV-LYON11	17	14.17
	Spinal cord biopsy	9 Oct	SHAV-LYON12	18.34	14.47
	Spleen biopsy	9 Oct	SHAV-LYON13	30.17	25.42
	Liver biopsy	9 Oct	SHAV-LYON14	28.71	25.8

ADIALYO SHLV: detection of Shamonda-like virus 2026; BAL: bronchoalveolar lavage; CSF: cerebrospinal fluid; Cq: quantification cycle; ND: not detected; SHAV: Shamonda virus; SHAV-EU1: detection of SHAV Europe 1 with RT-PCR.

Grey: samples from the upper and lower respiratory tract (bronchoalveolar lavage, throat, oral, saliva); Green: Cerebrospinal fluids and brain biopsies; Yellow: serum/plasma; White: other sample types.

Microbiological investigations of the CSF included multiplex PCR panels (Biofire, Biomerieux) for bacterial and viral pathogens, targeting, among others, *Cryptococcus*, herpes simplex virus (HSV) 1/2, cytomegalovirus (CMV), human herpesvirus (HHV)-6 and enterovirus. To avoid missing a pathogen due to limited sensitivity, independent pathogen-specific PCR assays targeting enterovirus, HHV-6, HSV and CMV were performed, along with other additional pathogen-specific PCR assays targeting HHV-8, Epstein–Barr virus (EBV), measles virus, parvovirus B19, human polyomavirus (JC virus) and adenovirus, with all results being negative. Further investigations ruled out tuberculosis, toxocariasis, toxoplasmosis and other bacterial and fungal infections through 16S rRNA gene and internal transcribed spacer (ITS) sequencing and culture. West Nile virus PCR on serum samples and IgG/IgM serological testing were also negative. Creutzfeldt–Jakob disease was subsequently ruled out.

We detected HSV in nasopharyngeal swabs, with positivity documented since 6 September, leading to initiation of foscarnet treatment due to acyclovir resistance.

## Detection by metagenomics and metatranscriptomics of Shamonda virus Europe 1

Given the negative results of the tests performed, two complementary next-generation sequencing (NGS) approaches were performed on the CSF sampled on D2. These methods involved viral metagenomics using an in-house protocol [7] which allows sequencing all extracellular viral particles and another in-house metatranscriptomic protocol which sequences total RNA present in a sample [8]. Detailed bioinformatics pipelines are available on https://github.com/genepii. Kraken2 results, obtained on D12, indicated 4,740 of 48 million reads and 1,694 of 30 million reads assigned to the genus *Orthobunyavirus* for the two methods, respectively. This was the main viral finding after the internal control (MS2 phage). No other pathogenic viruses, bacteria, fungi or parasites were identified. Importantly, all other samples and negative template controls processed alongside these runs were negative, ruling out any contamination. A total of 7 and 5 contigs obtained by de novo assembly (MetaSpades; https://github.com/ablab/spades) exhibited the greatest homology (93%) with the Shamonda virus RefSeq reference sequence (NC_018463, L segment). Blastn analysis (https://blast.ncbi.nlm.nih.gov/Blast.cgi) against the nt database (updated on 10 October) revealed that the contigs shared over 99% homology with SHAV-EU1 sequences released on GenBank (https://www.ncbi.nlm.nih.gov/genbank/) in August. After mapping the reads against SHAV-EU1 isolate TG_261216_18 sequences (PZ831165, PZ831161 and PZ831157, respectively for S, M and L segments), a partial genome was obtained (87% coverage for segment S, 65% for segment M and 71% for segment L coverage with sequencing depth of 10X), as presented in Supplementary Material 1.

Viral culture on cerebellum and spinal cord biopsies was performed on Vero E6 (ATCC CRL-1586; https://www.atcc.org) and revealed a cytopathogenic effect 3 days after inoculation. Long-read metatranscriptomics sequencing [9] confirmed SHAV-EU1 identity and allowed to retrieve a whole genome sequence (100% coverage) for each of the three segments. No difference was observed with the sequences obtained directly from CSF.

Phylogenetic analysis of the consensus whole genome obtained from the cerebellum biopsy, including the three segments, confirmed that the genome belonged to SHAV-EU1 clade, with no evidence of reassortment compared with SHAV-EU1 viruses isolated in animals (Figure 2). More details are presented in Supplementary Materials S1–S10. In total, 10 single nt polymorphisms (SNPs) were identified compared with Shamonda virus isolate TG_261216_18: four in segment L encoding the RNA-dependent RNA polymerase, five in segment M encoding the viral surface glycoproteins and one in segment S encoding the nucleocapsid. Most of these SNPs were non-synonymous, with two mutations in L (I900V, T1903I), four mutations in M (F504S, L574P, N716S, I1306V) and one mutation in S (G67S). Details are provided in Supplementary Material S9. Among these, five were specifically carried by the patient when compared with animal sequences. Whether these mutations might confer increased infection in human cells is currently unknown.

**Figure 2:**
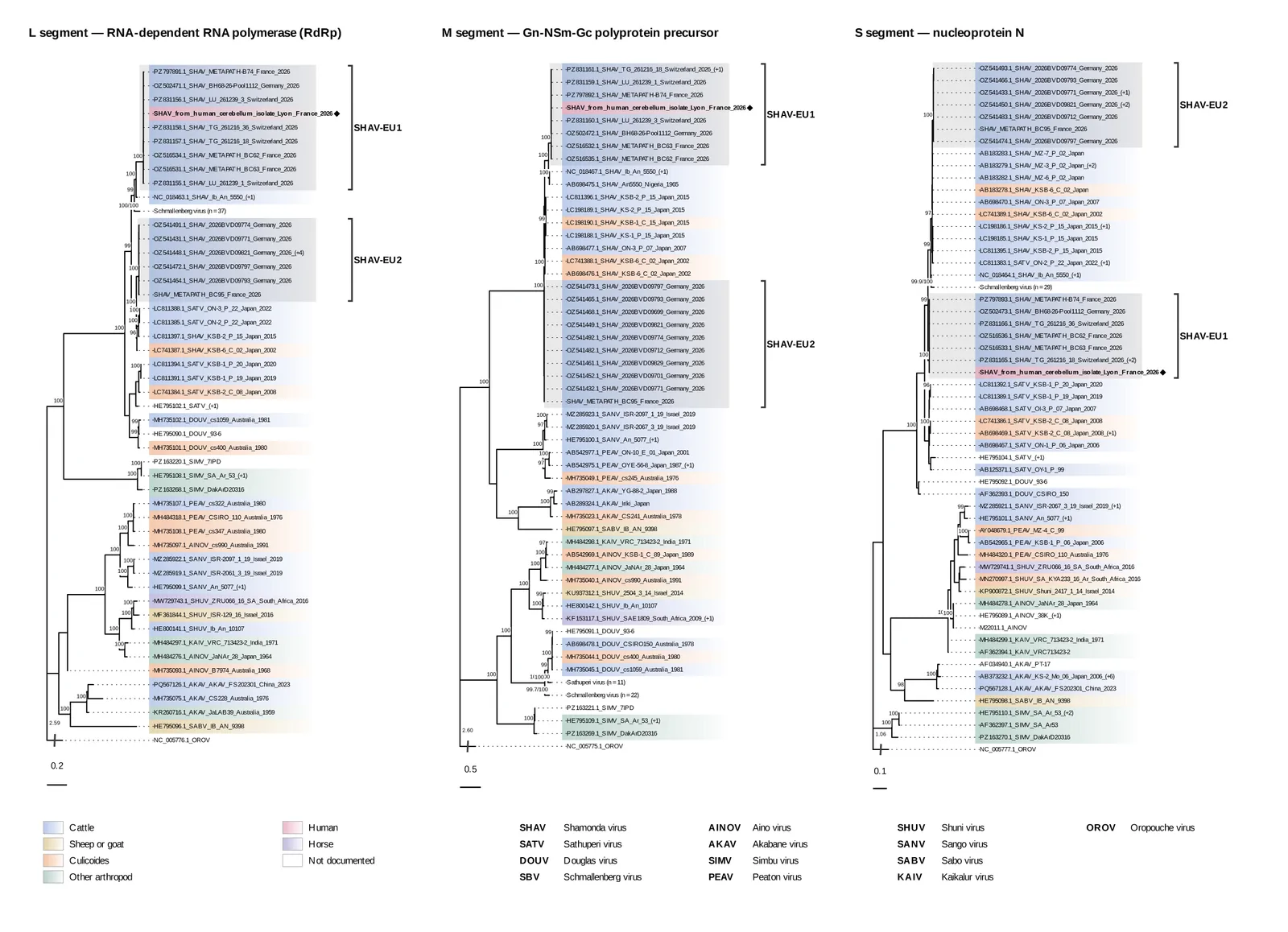
Maximum likelihood phylogenetic trees of the L, M and S genome segments of Shamonda virus Europe 1 detected in a human patient, France, 2026 and representative Simbu serogroup Orthobunyaviruses, 1965–2026

Branch support was assessed using 1,000 ultrafast bootstrap and 1,000 SH-aLRT replicates; a value is shown only where SH-aLRT is 80 or more and ultrafast bootstrap is 95 or more, and the value shown is the ultrafast bootstrap. The L, M and S trees included 80, 82 and 72 nt sequences, respectively. The strain from the patient is labelled in bold and marked by a diamond, and square brackets mark the two lineages of the 2026 European emergence, SHAV-EU1 and SHAV-EU2. The Schmallenberg virus clade of each segment, and the Sathuperi virus clade of the M segment, are each reduced to one line giving the virus name and the number of accessions it stands for. The tint behind each label gives the host of the sample; the key and the species abbreviations are at the foot of the figure. Oropouche virus, used as the outgroup, is shown at the foot of each panel with its branch cut (//) and its true length printed beside the cut. Scale bars are in substitutions per site, one per panel. The same three trees with every sequence labelled, for this specimen and for the cerebrospinal fluid consensus, are given in Supplementary Material.

The sequences were downloaded from GenBank.

We confirmed SHAV-EU1 positivity by RT-PCR on an independent aliquot of the CSF sampled on D0 (23 September). The RT-PCR assays were performed by the École Nationale Vétérinaire de Toulouse, using two protocols that both detected SHAV-EU1: one in-house assay, and the commercial ADIALYO SHLV kit (Bio-X Diagnostics, Rochefort, Belgium) (quantification cycle (Cq) = 33). The methods and all Cq values are presented in the Table and Supplementary Material S11. Notably, all four positive post-mortem biopsies from the central nervous system (cerebellum, spinal cord, frontal lobe and occipital lobe) exhibited very high viral loads (Cq < 15), as did the liver and spleen samples (Cq = 25).

## Retrospective investigation

We retrospectively screened samples from the patient that were available from the hospital by RT-PCR, with the oldest sample tested taken on 2 July (D−83) (Figure 1) and detected RNA of SHAV-EU1 in samples from stools, urine and saliva collected on D14 with Cq values of 29, 32 and 24, respectively. Respiratory samples, including lower and upper tract, were also positive for RNA SHAV-EU1 up to 3 weeks before the HSCT. A bronchoalveolar lavage, sampled in the context of a *Legionella* infection, was already positive (Cq  =  35) 2 months before symptom onset (21 July, D−64). Importantly, sera systematically tested positive for SHAV-EU1 with Cq ranging from 25 to 35 from 2 July (D−83) to D1. Subsequent blood tests were found negative. Finally, SHAV-EU1 was not detected in the graft, which, combined with the detection of the virus in the patient from early July, allows the graft to be ruled out as a route of transmission. All RT-PCR data are provided in the Table.

## Discussion

Here, we report the first human case of SHAV-EU1 infection, an emerging *Orthobunyavirus* that spread over Europe in summer 2026 affecting predominantly cattle. The patient, who had a severely weakened immune system, died from multi-organ failure including neuro-invasive disease, with primary neurological symptoms appearing on 23 September.

Investigations into the viral course revealed that the virus was present in the patient's blood for several weeks. At this point, we cannot date the time of infection as the earliest sample investigated was already positive for the virus on 2 July. Given that the first detections in cattle were reported in early July and clinical signs were documented in dairy cattle as early as June 2026, the patient was probably infected between mid-June and 1 July 2026. Investigation of the patient’s clinical history revealed that they developed an episode of diarrhoea and anorexia on 19 June, similar to symptoms observed in adult cattle [2] and could correspond to the first symptoms of this infection. Investigations on earlier blood and available respiratory samples are ongoing to determine the first detection of the virus over the clinical course by RT-PCR as well as by genomic investigation.

Route of transmission also remains unknown at this point. Graft transmission has been ruled out due to the negative PCR test of the graft and the previous positive PCR of the blood sample. Three different transmission hypotheses are currently investigated. As observed in animals, the most likely is the vector-borne transmission, presumably *Culicoides* spp. biting midges near the patient's home located in a rural area where animal transmission of SHAV-EU1 was officially documented in early July 2026, although transmission was likely already occurring in June 2026. The potential involvement of alternative arthropod vectors should also be explored. The two other routes (transfusion and food-borne) are less likely, given the sequence of events, and are not supported by any evidence at this point although investigations are ongoing to rule them out. Transfusion-related transmission would require an immunocompetent donor with asymptomatic viraemia in June, while viraemia is reported very transient in animals. Transmission by raw milk or raw cheese appears unlikely, as the presence of the virus in milk has not yet been reported and there is currently no evidence of direct contact-mediated transmission between animals.

The neurological presentation of this case is consistent with that described in horses infected with SHAV-EU [10,11], but also with Oropouche virus (OROV), the closest Orthobunyaviruses to infect humans. Oropouche virus primarily causes an acute febrile disease with diverse clinical manifestations, including neurological manifestations [12]. Other Orthobunyaviruses have been reported to cause severe encephalitis in patients with weakened immune systems [13,14]. As previously shown, this case highlights the importance of unbiased metagenomic methods for identifying uncharacterised infections in patients with weakened immune systems. Furthermore, the 1-week interval between the clinical request for NGS analysis and RT-PCR confirmation by the veterinary laboratory highlights the effective coordination between reference laboratories in France.

At this point, there is no evidence of other human infections while 53 of 96 departments of metropolitan France have confirmed cattle outbreaks in France as of 15 October [5]. Given the severe immunosuppression of the patient, SHAV-EU1 may have been able to infect the human host and to spread in multiple compartments (as indicated by positive findings in samples from central nervous system, stools and respiratory tract) despite low viral fitness in humans in general. Notably, high viral loads were reported in this patient's samples compared with those of infected animals [11], with viral culture positive in tissues and showing a cytopathogenic effect. High viral loads have been described in the brain of bovine fetuses [2], highlighting the neurotropic potential of this virus and the importance of immune competence in controlling viral dissemination. Molecular non-synonymous mutations, notably those reported in the spike protein, need to be carefully explored to understand the risk of human adaptation.

Further investigations, including anatomopathology, molecular compartmentalisation, assessment of viral infectivity and humoral immune response will help to understand the pathophysiology in this human case. We could not investigate the humoral response of this patient to SHAV-EU1 infection, as no commercial assay is currently available. Seroneutralisation assays will be implemented to further characterise the humoral immune response to this infection, although the patient’s weakened immune system may limit the interpretability of the results. In addition, these assays will be used to investigate potential exposure among family members and in the French population using available serum samples.

## Conclusion

We present the first case of human infection by the Shamonda-Europe virus, specifically the clade EU1, which emerged in European animals in June 2026. However, given the patient’s significantly weakened immune system, this finding alone cannot currently be interpreted as a serious risk for the general immunocompetent population. The primary hypothesis of transmission remains *Culicoides* biting midges and although direct human-to-human transmission appears unlikely, transmission through blood transfusion or organ transplantation remains a potential concern. Further investigations are warranted to better characterise transmission routes, particularly potential vector-borne transmission. This case calls for an enhanced surveillance of Shamonda Europe virus in both human and animal settings in Europe.

## Data Availability

Whole genome sequencing data have been deposited on Genbank under the following definitions: Shamonda virus human CSF isolate/HCL-France/2026 and Shamonda virus human cerebellum culture isolate/HCL-France/2026, corresponding to CSF and cerebellum isolate, respectively. Data are available upon reasonable request by contacting the corresponding authors. Bioinformatic pipelines implemented for viral metagenomics and metatranscriptomics analyses are available on github (https://github.com/genepii).

## Supplementary Data

4022000 Supplementary Material
