# Supplementary material for: Fatal encephalitis caused by Shamonda virus Europe 1 (SHAV-EU1) in a patient with a severely weakened immune system, France, 2026
Source: Euro Surveill. 2026 Oct 22;31(42):2600883. doi: 10.2807/1560-7917.ES.2026.31.42.2600883 (PMC13695005; doi:10.2807/1560-7917.ES.2026.31.42.2600883)
Supplement: Supplementary file 1 [file 26-00883_DESTRAS_Supplement.pdf]

Title: Fatal encephalitis caused by Shamonda-Europe virus in a severely immunocompromised patient, France, 2026

This supplementary material is hosted by *Eurosurveillance as supporting information alongside the article "**Fatal encephalitis caused by Shamonda-Europe virus in a severely immunocompromised patient, France, 2026**", on behalf of the authors, who remain responsible for the accuracy and appropriateness of the content. The same standards for ethics, copyright, attributions and permissions as for the article apply. Supplements are not edited by* Eurosurveillance and the journal is not responsible for the maintenance of any links or email addresses provided therein.

Supplementary Figure S1: Genome coverage of the patient's specimens, measured against the reference used to call the consensus sequences.

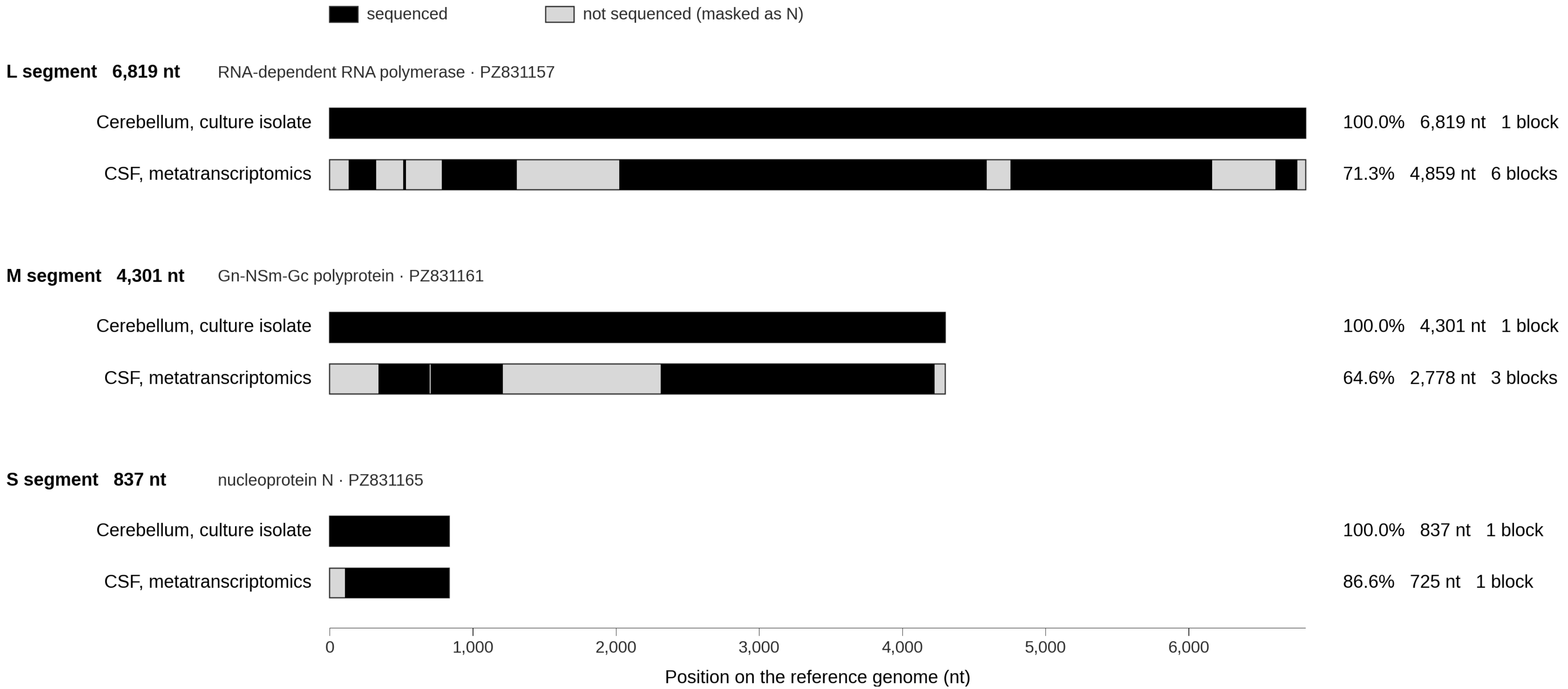

Coverage is measured against the Swiss deposit TG_261216_18 (PZ831157, PZ831161 and PZ831165 for the L, M and S segments), the reference on which both consensus sequences were called. Consensus were generated with a minimum of 10X sequencing depth. Abbreviations as in Figure S1.

Supplementary Figure S2: Segment by segment percent nucleotide identity of two sequences from the central nervous system of the patient with fatal encephalitis against SHAV-EU1 and SHAV-EU2 reference genomes.

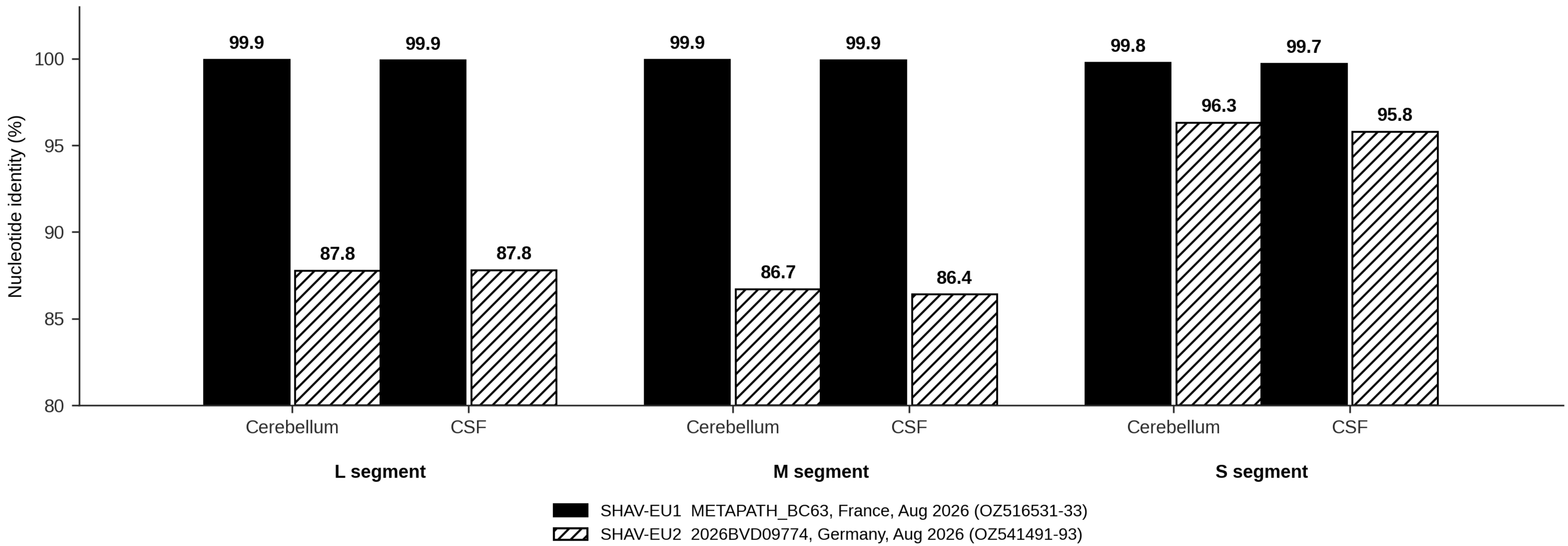

Two specimens from the same patient were compared: the sequence of a cerebellum culture isolate that was grown in cell culture from a cerebellum biopsy and a CSF metatranscriptomic consensus sequence that was read directly from the cerebrospinal fluid (CSF). Both reference genomes were held in a single index, so the aligner had to choose between them rather than being told which one to use. The alignment was done with minimap2 2.30, using the following options: -x asm20 (preset for assembly-to-reference alignment of sequences up to about 5% divergent), -c (compute the full base-level alignment and its CIGAR string), and --secondary=no (report only the best alignment of each query, so that one sequence cannot be counted on both references). Only the blocks aligned on both reference genomes were counted so that the two columns of a pair shared the same denominator and the comparison was not biased by the parts of the genome that were missing from one specimen. The two reconstructed genomes differ at 4 of 8,362 comparable positions, which corresponds to 99.95% identity. Abbreviations: CSF, cerebrospinal fluid, SHAV-EU1 and SHAV-EU2, the two lineages of the 2026 European Shamonda virus emergence.

Supplementary Figure S3: Maximum likelihood phylogenies of the three genome segments of Shamonda virus, with every sequence of the panel labelled. The same three trees as the main-text figure, drawn here without collapsing any clade, so that every sequence appears with its own label.

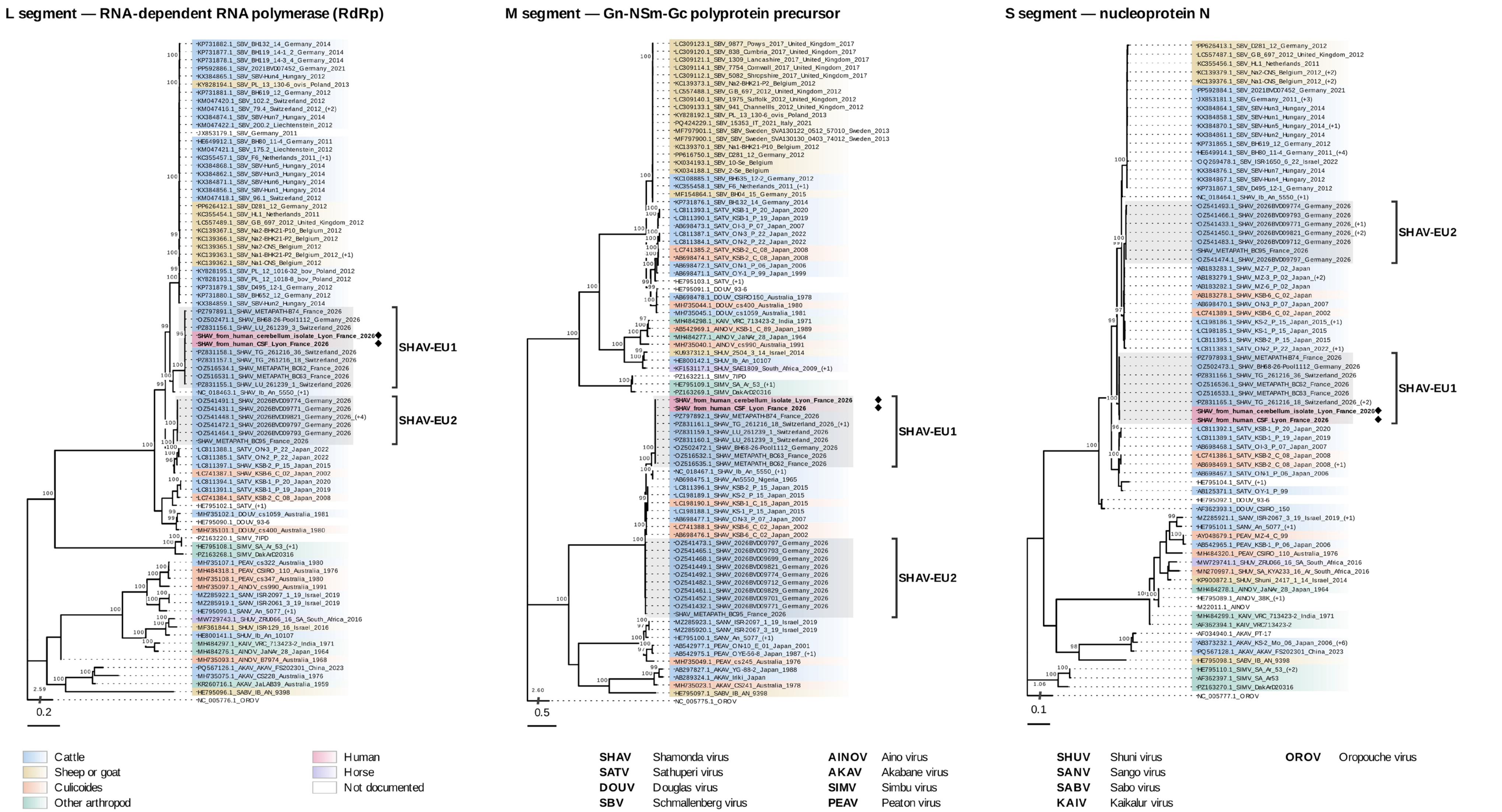

Methods and reading keys:

- Panel: All sequences of the Simbu serogroup available in GenBank for the segment concerned, one tree per segment. Oropouche virus was used as the outgroup to root each tree, then pruned from the display so that the ingroup fills the figure.

- Alignment: MAFFT 7.525 in L-INS-i mode (--localpair --maxiterate 1000), which refines the alignment iteratively from local pairwise alignments. This mode was chosen over the default progressive heuristic because the panel mixes complete genomes with partial sequences, a situation in which the fast mode misplaces the ends of the shorter sequences; on these three segments --auto selects L-INS-i for S but the faster FFT-NS-i for L and M.
- Tree inference: IQ-TREE 2.3.6 under the substitution model selected by ModelFinder on the Bayesian information criterion (-m MFP): GTR+F+I+R3 for L, GTR+F+I+G4 for M, TPM2u+F+G4 for S. GTR, general time-reversible; TPM2u, unequal-frequency three-parameter model; +F, empirical base frequencies; +I, a proportion of invariable sites; +G4, gamma-distributed rate heterogeneity in four categories; +R3, free-rate heterogeneity in three categories.
- Branch support: 1,000 ultrafast bootstrap replicates (-B 1000 --bnni) and 1,000 SH-aLRT replicates (--alrt 1000). One thousand replicates is the smallest number for which the authors of the method report the UFBoot value to be unbiased; --bnni re-optimises each replicate tree by nearest-neighbour interchange, which removes the residual optimism of the approximation. Reading the support. A branch is annotated only where SH-aLRT reaches 80 and UFBoot reaches 95, the two thresholds recommended for these measures; the number printed is the UFBoot value. An unannotated branch is not necessarily wrong, but it is not supported by both measures.
- Lineage assignment: The three segments independently place the patient strain inside SHAV-EU1, each with the maximal ultrafast bootstrap value of 100 (SH-aLRT 100 on L, 100 on M, 89.5 on S). Every support value printed on these figures was checked against the tree file it comes from, so that none is added and none omitted.

Supplementary Table S4: Closest public sequences to the patient's L segment, which encodes the viral RNA-dependent RNA polymerase (RdRp). Ten best BLASTN hits per specimen, ranked by bit score, among the sequences free of publication embargo.

Specimen	Accession	Isolate	Origin	Identity (%)	Coverage (%)	Aligned (nt)	Mismatches	Gaps	E-value	Bit score
Cerebellum,	OZ516531.1	METAPATH_BC63	France, 2026-08-05	99.9	100	6,819	4	0	0.0	12,571
culture isolate	PZ831157.1	TG_261216_18	Switzerland, 07-2026	99.9	100	6,819	4	0	0.0	12,571
	OZ502471.1	BH68-26-Pool1112	Germany, 2026-07-24	99.9	100	6,819	5	0	0.0	12,565
	PZ831155.1	LU_261239_1	Switzerland, 07-2026	99.9	100	6,819	5	0	0.0	12,565
	PZ831156.1	LU_261239_3	Switzerland, 07-2026	99.9	100	6,819	5	0	0.0	12,565
	OZ516534.1	METAPATH_BC62	France, 2026-08-06	99.9	100	6,819	7	0	0.0	12,554
	PZ797891.1	METAPATH-B74	France, 25-07-2026	99.9	99	6,804	7	0	0.0	12,526
	PZ831158.1	TG_261216_36	Switzerland, 07-2026	99.9	99	6,795	4	0	0.0	12,526
	HE795105.1	Ib An 5550	Prototype SHAV strain*	93.0	100	6,819	476	0	0.0	9,956
	NC_018463.1	Ib An 5550	Prototype SHAV strain*	93.0	100	6,819	476	0	0.0	9,956
Cerebro Spinal Fluid,	OZ502471.1	BH68-26-Pool1112	Germany, 2026-07-24	99.9	71	2,564	2	0	0.0	4,724
metatranscriptomics	PZ831155.1	LU_261239_1	Switzerland, 07-2026	99.9	71	2,564	2	0	0.0	4,724
	PZ831156.1	LU_261239_3	Switzerland, 07-2026	99.9	71	2,564	2	0	0.0	4,724
	PZ831157.1	TG_261216_18	Switzerland, 07-2026	99.9	71	2,564	2	0	0.0	4,724
	PZ831158.1	TG_261216_36	Switzerland, 07-2026	99.9	71	2,564	2	0	0.0	4,724
	OZ516531.1	METAPATH_BC63	France, 2026-08-05	99.9	71	2,564	3	0	0.0	4,719
	OZ516534.1	METAPATH_BC62	France, 2026-08-06	99.9	71	2,564	3	0	0.0	4,719
	PZ797891.1	METAPATH-B74	France, 25-07-2026	99.8	71	2,564	4	0	0.0	4,713
	HE795105.1	Ib An 5550	Prototype SHAV strain*	93.3	71	2,564	172	0	0.0	3,783
	NC_018463.1	Ib An 5550	Prototype SHAV strain*	93.3	71	2,564	172	0	0.0	3,783

* Prototype SHAV strain isolated in Nigeria and reported in 1965.

Supplementary Table S5: Closest public sequences to the patient's M segment, which encodes the Gn-NSm-Gc polyprotein precursor.
Ten best BLASTN hits per specimen, ranked by bit score, among the sequences free of publication embargo.

Specimen	Accession	Isolate	Origin	Identity (%)	Coverage (%)	Aligned (nt)	Mismatches	Gaps	E-value	Bit score
Cerebellum,	OZ502472.1	BH68-26-Pool1112	France, 2026-08-05	99.9	100	4,301	3	0	0.0	7,926
culture isolate	OZ516532.1	METAPATH_BC63	Switzerland, 07-2026	99.9	100	4,301	3	0	0.0	7,926
	PZ831159.1	LU_261239_1	Germany, 2026-07-24	99.9	100	4,301	3	0	0.0	7,926
	PZ831160.1	LU_261239_3	Switzerland, 07-2026	99.9	100	4,301	3	0	0.0	7,926
	PZ797892.1	METAPATH-B74	Switzerland, 07-2026	99.9	100	4,301	4	0	0.0	7,921
	PZ831161.1	TG_261216_18	France, 2026-08-06	99.9	100	4,301	5	0	0.0	7,915
	PZ831162.1	TG_261216_36	France, 25-07-2026	99.9	100	4,301	5	0	0.0	7,915
	OZ516535.1	METAPATH_BC62	Switzerland, 07-2026	99.9	99	4,275	3	0	0.0	7,878
	HE795106.1	Ib An 5550	Prototype SHAV strain*	92.6	100	4,301	316	1	0.0	6,185
	NC_018467.1	Ib An 5550	Prototype SHAV strain*	92.6	100	4,301	316	1	0.0	6,185
Cerebro Spinal Fluid,	OZ502472.1	BH68-26-Pool1112	Germany, 2026-07-24	99.9	65	1,915	2	0	0.0	3,526
metatranscriptomics	OZ516532.1	METAPATH_BC63	Switzerland, 07-2026	99.9	65	1,915	2	0	0.0	3,526
	PZ831159.1	LU_261239_1	Switzerland, 07-2026	99.9	65	1,915	2	0	0.0	3,526
	PZ831161.1	TG_261216_18	Switzerland, 07-2026	99.9	65	1,915	2	0	0.0	3,526
	PZ831162.1	TG_261216_36	Switzerland, 07-2026	99.9	65	1,915	2	0	0.0	3,526
	OZ516535.1	METAPATH_BC62	France, 2026-08-05	99.8	65	1,915	3	0	0.0	3,520
	PZ797892.1	METAPATH-B74	France, 2026-08-06	99.8	65	1,915	3	0	0.0	3,520
	PZ831160.1	LU_261239_3	France, 25-07-2026	99.8	65	1,915	3	0	0.0	3,520
	HE795106.1	Ib An 5550	Prototype SHAV strain*	92.6	65	1,915	141	0	0.0	2,756
	NC_018467.1	Ib An 5550	Prototype SHAV strain*	92.6	65	1,915	141	0	0.0	2,756

* Prototype SHAV strain isolated in Nigeria and reported in 1965.

Supplementary Table S6: Closest public sequences to the patient's S segment, which encodes the nucleoprotein N. Ten best BLASTN hits per specimen, ranked by bit score, among the sequences free of publication embargo.

Specimen	Accession	Isolate	Origin	Identity (%)	Coverage (%)	Aligned (nt)	Mismatches	Gaps	E-value	Bit score
Cerebellum,	PZ831163.1	LU_261239_1	France, 2026-08-05	99.9	100	837	1	0	0.0	1,541
culture isolate	PZ831164.1	LU_261239_3	Switzerland, 07-2026	99.9	100	837	1	0	0.0	1,541
	PZ831165.1	TG_261216_18	Germany, 2026-07-24	99.9	100	837	1	0	0.0	1,541
	OZ502473.1	BH68-26-Pool1112	Switzerland, 07-2026	99.8	100	837	2	0	0.0	1,535
	PZ831166.1	TG_261216_36	Switzerland, 07-2026	99.8	99	833	2	0	0.0	1,528
	OZ516536.1	METAPATH_BC62	France, 2026-08-06	99.9	99	829	1	0	0.0	1,526
	OZ516533.1	METAPATH_BC63	France, 25-07-2026	99.8	99	829	2	0	0.0	1,520
	HE795107.1	Ib An 5550	Switzerland, 07-2026	97.4	99	834	21	1	0.0	1,417
	NC_018464.1	Ib An 5550	Prototype SHAV strain*	97.4	99	834	21	1	0.0	1,417
	PZ797893.1	METAPATH-B74	Prototype SHAV strain*	99.9	91	760	1	0	0.0	1,399
Cerebro Spinal Fluid,	OZ502473.1	BH68-26-Pool1112	Germany, 2026-07-24	99.9	87	725	1	0	0.0	1,334
metatranscriptomics	PZ831163.1	LU_261239_1	Switzerland, 07-2026	99.9	87	725	1	0	0.0	1,334
	PZ831164.1	LU_261239_3	Switzerland, 07-2026	99.9	87	725	1	0	0.0	1,334
	PZ831165.1	TG_261216_18	Switzerland, 07-2026	99.9	87	725	1	0	0.0	1,334
	PZ831166.1	TG_261216_36	Switzerland, 07-2026	99.9	87	725	1	0	0.0	1,334
	OZ516536.1	METAPATH_BC62	France, 2026-08-05	99.9	86	722	1	0	0.0	1,328
	PZ797893.1	METAPATH-B74	France, 2026-08-06	99.9	86	720	1	0	0.0	1,325
	OZ516533.1	METAPATH_BC63	France, 25-07-2026	99.7	86	722	2	0	0.0	1,323
	HE795107.1	Ib An 5550	Prototype SHAV strain*	97.1	87	725	21	0	0.0	1,223
	NC_018464.1	Ib An 5550	Prototype SHAV strain*	97.1	87	725	21	0	0.0	1,223

* Prototype SHAV strain isolated in Nigeria and reported in 1965.

Supplementary Table S7: Nucleotide and amino acid sequence identity values for the L segment (RNA-dependent RNA polymerase, RdRp). Percent nucleotide identity below the diagonal, percent amino acid identity above it.

	**LYON**[a]	METAPATH BC63[b]	TG_261216 18[c]	BH68-26 Pool1112[d]	METAPATH BC95[e]	2026BV D09771[f]	SHAV	SATV	DOUV	SBV	AINOV	AKAV	SIMV	PEAV	SHUV	SANV
**LYON**[a]		**99.9**	**99.9**	**99.9**	**97.5**	**97.6**	**98.5**	**95.2**	**94.3**	**97.5**	**68.7**	**70.9**	**69.6**	**68.4**	**68.5**	**69.1**
METAPATH BC63[b]	**99.9**		100	100	97.6	97.7	98.6	95.3	94.3	97.6	68.7	70.9	69.7	68.4	68.5	69.1
TG_261216 18[c]	**99.9**	100		100	97.6	97.7	98.6	95.3	94.3	97.6	68.7	70.9	69.7	68.4	68.5	69.1
BH68-26 Pool1112[d]	**99.9**	100	100		97.6	97.7	98.6	95.3	94.3	97.6	68.7	70.9	69.7	68.4	68.5	69.1
METAPATH BC95[e]	**87.7**	87.8	87.7	87.7		99.9	98.3	95.7	94.5	97.8	68.7	70.5	69.5	68.4	68.5	69.1
2026BV D09771[f]	**87.7**	87.8	87.7	87.7	99.8		98.4	95.7	94.6	97.9	68.7	70.6	69.5	68.5	68.6	69.1
SHAV	**93.0**	93.1	93.1	93.1	90.1	90.2		95.8	94.5	98.4	68.8	70.9	69.8	68.5	68.5	69.2
SATV	**84.4**	84.4	84.4	84.4	84.4	84.4	84.9		96.2	95.9	68.4	71.0	70.4	68.4	68.5	68.8
DOUV	**84.3**	84.3	84.3	84.3	84.1	84.1	84.6	87.1		94.9	68.7	70.5	70.2	68.6	68.5	68.9
SBV	**89.3**	89.4	89.4	89.4	89.0	89.0	93.0	84.5	84.6		69.0	71.0	70.0	69.0	68.7	69.4
AINOV	**66.6**	66.6	66.6	66.6	66.3	66.3	66.0	66.4	66.4	66.8		68.2	71.3	88.7	95.2	90.2
AKAV	**67.2**	67.2	67.2	67.2	66.8	66.8	67.1	67.4	67.1	67.4	66.4		70.2	68.5	68.2	68.3
SIMV	**67.2**	67.2	67.2	67.2	67.3	67.4	67.3	67.5	67.7	67.7	68.2	67.5		71.0	71.2	71.1
PEAV	**66.0**	66.0	66.0	66.0	66.2	66.2	65.9	66.4	66.3	67.0	78.4	66.0	67.6		88.9	93.9
SHUV	**66.0**	66.0	66.0	66.0	66.6	66.7	66.2	65.8	66.6	66.1	85.0	65.7	67.9	77.9		90.5
SANV	**66.6**	66.6	66.6	66.6	67.1	67.0	66.7	66.8	66.3	66.9	79.2	65.9	68.3	84.8	78.7	

Computed on the coding sequence only, from pairwise global alignments guided by codons: each pair is aligned with EMBOSS needle 6.6.0 (Needleman-Wunsch global alignment, gap opening penalty 10, gap extension penalty 0.5), and a codon is counted only when it is resolved in both sequences, so that undetermined positions do not inflate or deflate the value.
(a) LYON, cerebellum culture strain isolated in Lyon of the patient (this study).
One complete genome per depositing laboratory of the 2026 European emergence: (b) METAPATH_BC63 (ENVT/INRAE, France, OZ516531-33), lineage SHAV-EU1; (c) TG_261216_18 (IVI, Switzerland, PZ831157/61/65), SHAV-EU1; (d) BH68-26-Pool1112 (FLI, Germany, OZ502471-73), SHAV-EU1; (e) METAPATH_BC95 (ENVT/INRAE, France, this study, not yet deposited), SHAV-EU2; (f) 2026BVD09771 (FLI, Germany, OZ541431-33), SHAV-EU2.
GenBank accession numbers: SHAV (Shamonda virus, NC_018463), SATV (Sathuperi virus, NC_018461), DOUV (Douglas virus, HE795090), SBV (Schmallenberg virus, NC_043583), AINOV (Aino virus, NC_018465), AKAV (Akabane virus, NC_009894), SIMV (Simbu virus, NC_018476), PEAV (Peaton virus, NC_043550), SHUV (Shuni virus, NC_043699), SANV (Sango virus, NC_043556).

Supplementary Table S8: Nucleotide and amino acid sequence identity values for the M segment (Gn-NSm-Gc polyprotein precursor).

Percent nucleotide identity below the diagonal, percent amino acid identity above it.

	**LYON[a]**	METAPATH BC63[b]	TG_261216 18[c]	BH68-26 Pool1112[d]	METAPATH BC95[e]	2026BV D09771[f]	SHAV	SATV	DOUV	SBV	AINOV	AKAV	SIMV	PEAV	SHUV	SANV
**LYON[a]**		**99.9**	**99.7**	**99.9**	**94.5**	**94.6**	**97.5**	**39.3**	**39.6**	**38.9**	**38.6**	**49.1**	**41.6**	**48.9**	**39.5**	**49.4**
METAPATH BC63[b]	**99.9**		99.9	100.0	94.7	94.7	97.7	39.4	39.6	38.9	38.7	49.2	41.6	48.8	39.5	49.4
TG_261216 18[c]	**99.9**	99.9		99.9	94.5	94.6	97.5	39.5	39.8	38.9	38.7	49.2	41.4	48.8	39.0	49.4
BH68-26 Pool1112[d]	**100.0**	100.0	99.9		94.7	94.7	97.7	39.4	39.6	38.9	38.7	49.2	41.6	48.8	39.5	49.4
METAPATH BC95[e]	**86.6**	86.6	86.6	86.7		99.9	95.3	39.2	39.8	38.9	38.8	49.9	41.6	49.3	39.5	49.7
2026BV D09771[f]	**86.6**	86.6	86.6	86.7	100.0		95.4	39.1	39.8	38.8	38.8	49.9	41.6	49.3	39.5	49.7
SHAV	**92.6**	92.6	92.6	92.7	88.8	88.8		39.3	39.4	39.2	38.5	49.4	41.4	49.1	39.0	49.6
SATV	**50.4**	50.4	50.3	50.4	51.1	51.1	50.3		91.6	90.2	58.3	39.2	51.5	41.8	57.1	41.0
DOUV	**50.4**	50.4	50.5	50.4	50.5	50.5	50.2	85.9		89.0	58.4	39.4	51.5	41.3	57.8	40.5
SBV	**49.7**	49.7	49.7	49.7	49.7	49.7	49.6	82.2	81.7		58.1	39.4	52.3	41.0	57.3	40.8
AINOV	**49.9**	49.9	49.9	49.9	49.5	49.5	49.2	60.4	61.0	60.6		39.6	53.8	39.5	75.9	39.6
AKAV	**54.5**	54.5	54.5	54.5	55.3	55.4	54.5	50.8	50.4	50.4	49.7		41.4	50.4	40.0	49.8
SIMV	**51.9**	51.9	51.8	51.9	52.1	52.1	51.8	57.5	57.8	57.6	59.5	50.3		41.2	52.2	41.8
PEAV	**55.4**	55.4	55.4	55.4	55.5	55.4	55.4	51.5	51.5	50.9	50.2	56.3	50.8		39.5	89.9
SHUV	**51.4**	51.4	51.0	51.4	50.8	50.8	50.6	60.9	60.8	61.2	71.6	50.2	59.3	50.8		40.4
SANV	**55.8**	55.8	55.8	55.8	56.5	56.5	56.0	51.7	51.8	50.5	50.5	56.7	51.9	83.9	51.7	

Computed on the coding sequence only, from pairwise global alignments guided by codons: each pair is aligned with EMBOSS needle 6.6.0 (Needleman-Wunsch global alignment, gap opening penalty 10, gap extension penalty 0.5), and a codon is counted only when it is resolved in both sequences, so that undetermined positions do not inflate or deflate the value.
(a) LYON, cerebellum culture strain isolated in Lyon of the patient (this study).
One complete genome per depositing laboratory of the 2026 European emergence: (b) METAPATH_BC63 (ENVT/INRAE, France, OZ516531-33), lineage SHAV-EU1; (c) TG_261216_18 (IVI, Switzerland, PZ831157/61/65), SHAV-EU1; (d) BH68-26-Pool1112 (FLI, Germany, OZ502471-73), SHAV-EU1; (e) METAPATH_BC95 (ENVT/INRAE, France, this study, not yet deposited), SHAV-EU2; (f) 2026BVD09771 (FLI, Germany, OZ541431-33), SHAV-EU2.
GenBank accession numbers: SHAV (Shamonda virus, NC_018463), SATV (Sathuperi virus, NC_018461), DOUV (Douglas virus, HE795090), SBV (Schmallenberg virus, NC_043583), AINOV (Aino virus, NC_018465), AKAV (Akabane virus, NC_009894), SIMV (Simbu virus, NC_018476), PEAV (Peaton virus, NC_043550), SHUV (Shuni virus, NC_043699), SANV (Sango virus, NC_043556).

Supplementary Table S9: Nucleotide and amino acid sequence identity values for the S segment (nucleoprotein N). Percent nucleotide identity below the diagonal, percent amino acid identity above it.

	**LYON**[a]	METAPATH BC63[b]	TG_261216 18[c]	BH68-26 Pool1112[d]	METAPATH BC95[e]	2026BV D09771[f]	SHAV	SATV	DOUV	SBV	AINOV	AKAV	SIMV	PEAV	SHUV	SANV
**LYON**[a]		**99.6**	**99.6**	**99.6**	**99.1**	**99.1**	**99.1**	**99.6**	**99.1**	**99.1**	**79.8**	**80.3**	**79.8**	**80.7**	**80.2**	**80.7**
METAPATH BC63[b]	**99.7**		100	100	99.6	99.6	99.6	100.0	99.6	99.6	80.3	80.3	80.3	81.1	80.6	81.1
TG_261216 18[c]	**99.9**	99.9		100	99.6	99.6	99.6	100.0	99.6	99.6	80.3	80.3	80.3	81.1	80.6	81.1
BH68-26 Pool1112[d]	**99.9**	99.9	100.0		99.6	99.6	99.6	100.0	99.6	99.6	80.3	80.3	80.3	81.1	80.6	81.1
METAPATH BC95[e]	**95.7**	95.7	95.8	95.8		100	100	99.6	99.1	100	79.8	79.8	80.3	80.7	80.2	80.7
2026BV D09771[f]	**95.7**	95.7	95.8	95.8	100		100	99.6	99.1	100	79.8	79.8	80.3	80.7	80.2	80.7
SHAV	**97.0**	97.0	97.1	97.1	97.6	97.6		99.6	99.1	100	79.8	79.8	80.3	80.7	80.2	80.7
SATV	**94.3**	94.3	94.4	94.4	93.0	93.0	93.0		99.6	99.6	80.3	80.3	80.3	81.1	80.6	81.1
DOUV	**93.6**	93.6	93.7	93.7	92.1	92.1	93.0	93.0		99.1	80.3	80.3	80.7	81.1	80.6	81.1
SBV	**95.4**	95.4	95.6	95.6	95.8	95.8	97.7	93.1	92.3		79.8	79.8	80.3	80.7	80.2	80.7
AINOV	**77.0**	77.0	77.1	77.1	76.5	76.5	76.7	78.2	78.4	77.0		82.8	83.3	99.1	99.6	99.1
AKAV	**77.8**	78.0	78.0	78.0	77.2	77.2	77.4	78.7	78.2	77.2	77.4		81.5	82.8	82.3	82.8
SIMV	**78.4**	78.4	78.5	78.5	78.5	78.5	78.7	79.3	80.5	78.7	78.5	78.8		83.3	83.2	83.3
PEAV	**77.0**	77.1	77.1	77.1	76.8	76.8	77.5	77.7	77.8	77.2	91.3	76.8	77.8		99.6	100
SHUV	**78.0**	78.0	78.2	78.2	77.7	77.7	77.9	78.3	78.5	77.9	94.0	76.3	78.2	91.7		99.6
SANV	**76.8**	77.0	77.0	77.0	76.5	76.5	77.4	78.1	78.4	78.2	91.3	77.7	77.5	92.6	91.1	

Computed on the coding sequence only, from pairwise global alignments guided by codons: each pair is aligned with EMBOSS needle 6.6.0 (Needleman-Wunsch global alignment, gap opening penalty 10, gap extension penalty 0.5), and a codon is counted only when it is resolved in both sequences, so that undetermined positions do not inflate or deflate the value.

(a) LYON, cerebellum culture strain isolated in Lyon of the patient (this study).

One complete genome per depositing laboratory of the 2026 European emergence: (b) METAPATH_BC63 (ENVT/INRAE, France, OZ516531-33), lineage SHAV-EU1; (c) TG_261216_18 (IVI, Switzerland, PZ831157/61/65), SHAV-EU1; (d) BH68-26-Pool1112 (FLI, Germany, OZ502471-73), SHAV-EU1; (e) METAPATH_BC95 (ENVT/INRAE, France, this study, not yet deposited), SHAV-EU2; (f) 2026BVD09771 (FLI, Germany, OZ541431-33), SHAV-EU2.

GenBank accession numbers: SHAV (Shamonda virus, NC_018463), SATV (Sathuperi virus, NC_018461), DOUV (Douglas virus, HE795090), SBV (Schmallenberg virus, NC_043583), AINOV (Aino virus, NC_018465), AKAV (Akabane virus, NC_009894), SIMV (Simbu virus, NC_018476), PEAV (Peaton virus, NC_043550), SHUV (Shuni virus, NC_043699), SANV (Sango virus, NC_043556).

Supplementary Table S10: The seven amino acid substitutions reported for the Lyon patient strain, read across all Shamonda virus strains with a complete genome. Amino acid carried at each of the seven positions, one strain per row. The row of the Lyon patient strain is in bold.

Strain	Country	Year	L_900	L_1903	M_504	M_574	M_716	M_1306	S_67
2026BVD09699 (SHAV-EU2)	Germany	2026	I	T	S	L	S	I	G
2026BVD09701 (SHAV-EU2)	Germany	2026	I	T	S	L	S	I	G
2026BVD09712 (SHAV-EU2)	Germany	2026	I	T	S	L	S	I	G
2026BVD09771 (SHAV-EU2)	Germany	2026	I	T	S	L	S	I	G
2026BVD09774 (SHAV-EU2)	Germany	2026	I	T	S	L	S	I	G
2026BVD09793 (SHAV-EU2)	Germany	2026	I	T	S	L	S	I	G
2026BVD09797 (SHAV-EU2)	Germany	2026	I	T	S	L	S	I	G
2026BVD09821 (SHAV-EU2)	Germany	2026	I	T	S	L	S	I	G
2026BVD09829 (SHAV-EU2)	Germany	2026	I	T	S	L	S	I	G
BH68-26-Pool1112 (SHAV-EU1)	Germany	2026	I	T	S	L	S	I	G
Ib An 5550	Nigeria	1965	I	T	S	L	S	I	G
KSB-2/P/15	Japan	2015	I	T	S	L	S	I	G
KSB-6/C/02	Japan	2002	I	T	S	L	S	I	G
LU_261239_1 (SHAV-EU1)	Switzerland	07-2026	I	T	S	L	S	I	G
LU_261239_3 (SHAV-EU1)	Switzerland	07-2026	I	T	S	L	S	I	G
**Lyon strain, this study (SHAV-EU1)**	France	2026	**V**	**I**	**S**	**P**	**S**	**V**	**S**
METAPATH-B74 (SHAV-EU1)	France	25-07-2026	I	T	S	L	S	I	G
METAPATH_BC62 (SHAV-EU1)	France	2026	I	T	S	L	S	I	G
METAPATH_BC63 (SHAV-EU1)	France	2026	I	T	S	L	S	I	G
TG_261216_18 (SHAV-EU1)	Switzerland	07-2026	I	T	F	L	N	I	G
TG_261216_36 (SHAV-EU1)	Switzerland	07-2026	I	T	F	L	N	I	G

Positions are numbered along the protein encoded by the segment named in the column header, counting from the first residue of the open reading frame (ORF). Residues are read through the multiple sequence alignment used for the phylogenies, so that insertions and deletions cannot shift the numbering. Only strains with all three segments deposited are shown.

Supplementary Methods S11. SHAV-EU1 RT-PCR assays

Sample preparation and nucleic acid extraction

Various types of samples were processed: tissues (frontal and occipital biopsy of the brain, cerebellum biopsy, spinal cord biopsy, liver and spleen biopsy) and other matrices [plasma, serum, saliva sample, urine sample, stools, bronchoalveolar lavage (BAL), cerebrospinal fluid (CSF), respiratory samples]. For tissue samples, approximately 40 mg were finely chopped using a scalpel, then transferred to a 2 ml tube containing 1 ml of sterile PBS. The sample was vortexed for 30 sec, then briefly centrifuged. For saliva and stools, samples were swabbed and the swab was resuspended in 500 µL of sterile PBS and vortexed for 5 min. A 150 µL aliquot of each pretreated sample was then used for extraction. All other samples were made up to 150 µl with sterile PBS. Nucleic acids were extracted using the NucleoSpin® RNA Virus (Macherey-Nagel, 740956.250) in accordance with the manufacturer’s protocol, with the exception of the elution step: the nucleic acids were eluted into 30 µl of RNase- and DNase-free water supplied with the kit, preheated to 70°C, and the eluate was passed through the column a second time to improve the yield. An extraction negative control, consisting of 150 µl of the PBS used for the sample pre-treatment, was processed in parallel with the samples. The eluates were used immediately for PCR, and the remainder was stored at −80°C.

Real-time PCR assay

Two real-time PCR tests were used. The first was carried out using the commercial ADIALYO SHLV kit (Bio-X, Rochefort, Belgium; ref: ADL82Y1-100) in accordance with the supplier’s recommendations: 5 µL of nucleic acid extract was added to 10 µL of reaction mixture. The second test was carried out using the iTaq Universal Probes One-Step Kit (BIO-RAD, 1725140) with the in-house SHAV-EU1 design: 2 µl of nucleic acid extract was added to give a final reaction volume of 20 µl, in accordance with the supplier’s recommendations.

- SHAV-EU1-Forward: 5’ GCACAGGATTTTGCCCAAGG 3’
- SHAV-EU1-Reverse: 5’ ATTCTTCGCAGCCCCATGAA 3’
- SHAV-EU1-Probe: 5’ 6-FAM/CCCCATACT/ ZEN™/ACAAATTGGTTGACA/ IB™FQ 3’

Both tests were performed on a LightCycler® 96 (Roche) following the cycling conditions recommended by the manufacturers. Each run included the extraction negative control, a PCR control without template, and the positive control supplied with the commercial ADIALYO SHLV kit, as well as a viral production extract standard for the in-house test. The data were analysed using the LightCycler® 96 software (version 1.1.0.1320), and the results were expressed as quantification cycle (Cq) values.
